# Comparison of two integration methods for dynamic causal modeling of electrophysiological data

**DOI:** 10.1016/j.neuroimage.2018.02.031

**Published:** 2018-06

**Authors:** Jean-Didier Lemaréchal, Nathalie George, Olivier David

**Affiliations:** a Sorbonne Universités, UPMC Univ Paris 06, Inserm, CNRS, Institut du Cerveau et de la Moelle épinière (ICM), Hôpital Pitié-Salpêtrière, Boulevard de l'hôpital, F-75013, Paris, France; b Sorbonne Universités, UPMC Univ Paris 06 UMR S 1127, Inserm U 1127, CNRS UMR 7225, Institut du Cerveau et de la Moelle épinière, ICM, Ecole Normale Supérieure, ENS, Centre MEG-EEG, F-75013, Paris, France; c Inserm, U1216, F-38000, Grenoble, France; d Univ. Grenoble Alpes, Grenoble Institut des Neurosciences, GIN, F-38000 Grenoble, France

**Keywords:** DCM, Neuronal model, Delay differential equations, Conduction delays

## Abstract

Dynamic causal modeling (DCM) is a methodological approach to study effective connectivity among brain regions. Based on a set of observations and a biophysical model of brain interactions, DCM uses a Bayesian framework to estimate the posterior distribution of the free parameters of the model (*e.g.* modulation of connectivity) and infer architectural properties of the most plausible model (*i.e.* model selection). When modeling electrophysiological event-related responses, the estimation of the model relies on the integration of the system of delay differential equations (DDEs) that describe the dynamics of the system. In this technical note, we compared two numerical schemes for the integration of DDEs. The first, and standard, scheme approximates the DDEs (more precisely, the state of the system, with respect to conduction delays among brain regions) using ordinary differential equations (ODEs) and solves it with a fixed step size. The second scheme uses a dedicated DDEs solver with adaptive step sizes to control error, making it theoretically more accurate. To highlight the effects of the approximation used by the first integration scheme in regard to parameter estimation and Bayesian model selection, we performed simulations of local field potentials using first, a simple model comprising 2 regions and second, a more complex model comprising 6 regions. In these simulations, the second integration scheme served as the standard to which the first one was compared. Then, the performances of the two integration schemes were directly compared by fitting a public mismatch negativity EEG dataset with different models. The simulations revealed that the use of the standard DCM integration scheme was acceptable for Bayesian model selection but underestimated the connectivity parameters and did not allow an accurate estimation of conduction delays. Fitting to empirical data showed that the models systematically obtained an increased accuracy when using the second integration scheme. We conclude that inference on connectivity strength and delay based on DCM for EEG/MEG requires an accurate integration scheme.

## Introduction

Dynamic causal modeling (DCM) is a model-driven approach used to infer causal relationships between brain areas (Friston, 2011). Based upon a realistic local neuronal architecture and biophysical parameters, it gives the possibility to explain experimental data with a biophysical model of effective connectivity. This generative approach has been applied to different functional imaging modalities, including functional magnetic resonance imaging (fMRI), electroencephalography (EEG), magnetoencephalography (MEG) or local field potentials (LFP). The key features of DCM are to explain differences between conditions by modulations in effective connectivity across distant neuronal populations and to test hypotheses about model architecture (Penny et al., 2004) within multiple subjects using either fixed- or random-effect methods (Stephan et al., 2009).

In this technical note, we focus on DCM for event-related potentials (ERP) using electrophysiological data (LFP, MEG, and EEG). The time resolution of these modalities gives a precious insight into the neuronal dynamics occurring within a range of a few milliseconds. Neural mass models for ERP embody two neuronal mechanisms operating at comparable time scales, namely the synaptic and the axonal transmission (David and Friston, 2003). These two fundamental mechanisms, necessary for the propagation of the neuronal activity x(t) through the different neuronal populations of the model, lead to describe the temporal evolution of the system $\dot{x}\left(t\right)=dx\left(t\right)/dt$ as a system of differential equations with respect to time. Although most DCM studies of ERP focus on coupling strengths and their modulation between conditions, all the parameters of the neuronal model contribute to the response of the system. In particular, the conduction delays between regions and the synaptic time constants are parameters which have to be estimated precisely during the computational inversion of the DCM – similar considerations are also relevant for neural field models (Jirsa, 2009). For example, considering a simple ERP model where only one region influences another region, both the synaptic time constants and the conduction delay between the two regions will directly influence the latency and amplitude of the response peaks. In addition, when specifying simple models, all connections do not necessarily model direct pathways, because there can be unspecified relays, and the parameterization should thus allow a large range of values for conduction delays between regions.

The mathematical specification of the model rests on the use of time-delay differential equations (DDEs) to take into account the temporal dependencies of the system through the presence of various (extrinsic and intrinsic, synaptic and conduction) delays. The accuracy of the solution of the dynamical system and the inversion scheme (estimation of posterior parameters) therefore directly depends on the method used to integrate the DDEs. The solution chosen in the current implementation of DCM for ERP (SPM12, version r6732, www.fil.ion.ucl.ac.uk/spm) is to approximate the DDEs with ordinary differential equations (ODEs) using a first order Taylor approximation for conduction delays (David et al., 2006, Appendix A). This approximation assumes conduction delays small enough compared to the global temporal dynamics of the system. In this context, the inversion of the system reduces to the integration of a simple set of ODEs. In this note, we assess the effects of this approximation with respect to the sensibility of Bayesian model selection and the accuracy of parameter estimation. We also evaluate it by using a second integration scheme of DDEs, more precise though computationally more expensive.

First, we briefly introduce the temporal parameters and the system of DDEs used by the ERP neuronal model and describe the two integration schemes used to solve this system. In particular, we compare the effect of varying conduction delays on the response of the system for a simple model with 2 sources. Second, we test the two DDEs integration schemes in the context of DCM. We use simulations to generate data with various conduction delays and modulations of connection and focus on the capability to identify the generative model among a series of plausible models and to retrieve parameter estimates for connectivity as close as possible to those used to generate the data. Last, we illustrate the effects of the DDEs integration scheme on a public mismatch negativity (MMN) EEG dataset (available at www.fil.ion.ucl.ac.uk/spm/data/eeg_mmn). The MMN paradigm has been extensively studied with DCM, with a minimal network of five cortical sources that could account for the differences observed between evoked responses to frequent and rare auditory stimuli (Garrido et al., 2007). This public dataset was therefore a candidate of choice to extend the LFP simulations to a real EEG case study.

## Material and methods

### ERP neural model

The neural mass model used in this note is the ERP model composed of 3 neuronal sub-population and described in (David et al., 2006, Ostwald and Starke, 2016). Two sets of parameters are particularly important for the dynamics of the system. First, synaptic time constants (excitatory and inhibitory) control the temporal integration of afferent connections entering one neuronal population. Mathematically, they parameterize a convolution kernel, which transforms an average density of presynaptic inputs into an average postsynaptic membrane potential. Second, conduction delays represent the amount of time the output of one neuronal population needs to propagate to another neuronal population (along white matter fibers). A distinction is made between intrinsic (source and target neuronal populations belong to the same brain region) and extrinsic (source and target neuronal populations belong to distinct brain region) conduction delays. Although the individual impacts of these parameters on the dynamics are clear when a single population is considered, they are much more difficult to disentangle when the system is composed of multiple interconnected populations, as it is usually the case with DCM.

In DCM for ERP, synaptic time constants are usually set *a priori* to 8 ms and 16 ms for excitatory and inhibitory synapses, respectively. Intrinsic conduction delays are fixed for all regions (2 ms), while extrinsic conduction delays are free parameters estimated separately for each connection between distinct regions (using a prior of 16 ms). Taking into account of the synaptic and axonal propagation leads to a dynamical description of the system as a set of DDEs, relating the input, output and state variables of the system.

### Integration schemes of DDEs

Fitting a DCM model to EEG or MEG data proceeds as an iterative variational Bayes optimization scheme (Friston et al., 2007). To compute the estimates of the posterior parameters, this approach requires the system of DDEs to be integrated many times at each step. We start from the specification of the system of DDEs:

(1) $$\dot{x}\left(t\right)=\varphi_{\theta }\left(t,\;u\left(t\right),{\left(x\left(t-d_{kl}\right)\right)}_{1\leq k,l\leq \nu }\right)$$

where x(t) represents the global state of the system, *i.e.* all state variables modeling neuronal population states (current, voltage), as a function of time and $\dot{x}\left(t\right)$ its time derivative. $\varphi_{\theta }$ is the general evolution function of the system, u(t) is a stimulus input entering the system, $d_{kl}$ is the conduction delay from state l to state k and ν is the total number of states describing the system. Importantly, $d_{kl}$ is null when k and l are states of the same neuronal population. Otherwise, $d_{kl}$ refers to an *intrinsic* conduction delay when k and l are states of distinct neuronal populations from the same brain region and to an *extrinsic* conduction delay when k and l are states of distinct brain regions.

The current implementation of DCM for ERP (SPM12, version r6732) uses a 2-steps method for the integration of Eq. (1) (see (David et al., 2006) Appendix A.2 and (Ostwald and Starke, 2016) for a detailed review of the method). The first step uses the 1st order Taylor approximation ψ of $\varphi_{\theta }$ in the neighborhood of x(t) with respect to conduction delays to transform the system of DDEs into a system of ODEs:

(2) $$\dot{x}\left(t\right)=\psi \left(t,x\left(t\right)\right)$$

The second step uses a Taylor expansion of x(t) at t+τ, where τ represents the time step of integration and is chosen to be the sampling rate of the EEG/MEG data, to provide the iterative integration scheme:

(3) x(t+τ)=x(t)+Uψ(t,x(t))

In Eq. (3), $U=\left(exp\left(\tau J\right)-I\right)J^{-1}$ and J=∂ψ/∂x is the Jacobian of the system evaluated at x(t). This explicit integration scheme is computationally very efficient, yet it potentially suffers from two sources of inaccuracy: the 1st order Taylor approximation of $\varphi_{\theta }$ in its first step and the choice of a fixed step-size of integration τ along the whole time interval (shorter step-size would be desirable whenever the local temporal dynamics of the system speeds up).

An alternative to the above-described method for solving the system of DDEs in Eq. (1) is to use a well-established extension of standard Runge-Kutta techniques, which is classically used for integrating ODEs. For a typical uni-dimensional ODE of the following form:

(4) $$\dot{x}\left(t\right)=f\left(t,x\left(t\right)\right)$$

Runge-Kutta (RK) techniques use an iterative procedure (method of steps).

(5) $$x_{n+1}=x_{n}+h\sum_{i=1}^{s}b_{i}k_{i}$$

where *h* is a step size, *s* a number of stages in the step, $b_{i}$ are weights and $k_{i}=f\left(t_{n}+c_{i}h,x_{n}+h\sum_{j=1}^{K}a_{ij}k_{j}\right)$ are evaluations of the function at intermediate stages $c_{i}$ (*K*=*i-1* for explicit case and *K*=*s* for implicit case).

Extension of this method to the integration of DDEs in Eq. (1) consists in using explicit RK when optimal step size is smaller than the smallest delay (all needed delayed values are available from accurate continuous extensions of previous steps) and implicit RK when step size is greater than the smallest delay (delayed values are then inside the step and an additional linear system needs to be solved). Very importantly, the step size is chosen small enough to control the local truncation error (adaptive method) and large enough to make the computation efficient. Therefore, this method estimates a very accurate solution of the DDEs with respect to the delays of conduction and the local temporal dynamics of the system. The theory and properties of the numerical scheme used in this integration are reviewed in (Shampine and Thompson, 2001). These include the proof of its uniform convergence, the treatment of short delays and their effects on stability, and the control of the relative and absolute error. The adaptation to the current context of Eq. (1) is described in the Appendix. Based on previous description, we use the notations *DDE_TA* (1st order Taylor approximation with fixed step size) and *DDE_RK* (RK technique with adaptive step size) to refer to these two integration schemes throughout this note. From the preceding remarks, we started with the proposition that DDE_RK integrator was more accurate than DDE_TA to solve the system of DDEs presented in Eq. (1).

In an initial step, we generated datasets from a simple ERP model with 2 regions connected with a forward connection only, in order to examine the effect of the DDE_TA integration scheme on the generated data in comparison with DDE_RK; this was examined for conduction delays varying between 4 and 32 ms.

### Integrating DDEs for DCM

One important feature of DCM is the ability to characterize differences measured between experimental conditions in terms of modulations of extrinsic and/or intrinsic connectivity. Modulation of extrinsic connectivity refers to a change of connection strength between two brain areas whereas modulation of intrinsic connectivity refers to a local change of synaptic gain in one brain area of the neural mass model. Specific hypotheses about modulations can be embedded in a model through a specific architecture. Fitting a series of competitive models to sufficient amount of data then gives the possibility to compare their respective architecture, identify the most plausible hypothesis, and finally make inferences about the posterior estimates of connectivity parameters. In the following, this typical workflow of analysis serves as a guideline to assess the effects of using DDE_TA integration scheme on the estimation of effective connectivity in the DCM context, first with simulations and then with a real example dataset.

### Simulations

#### Data generation

To compare DDE_TA and DDE_RK methods, we generated data from the ERP neural mass model of DCM. Given this generative model and a set of parameters, each dataset was simulated in a baseline and a modulated condition. In the modulated condition, all forward and backward connection strengths were changed from their baseline respective values while other parameters stayed unchanged. Because of the theoretical reasons briefly mentioned in section 2.2 and detailed in (Shampine and Thompson, 2001), the DDE_RK integration scheme was used to generate the data and reproduce accurately the temporal dynamics described by the equations of the neural mass model. The model received a brief input, described by a Gaussian function. Conduction delays between regions were systematically specified and imposed to be identical between each pair of reciprocal connection. Observation noise was created by convolving Gaussian noise with a smoothing kernel of 4 time samples (corresponding to 4 ms duration). This observation noise was then scaled in order to get a ratio of the mean power of the simulated signal over the mean power of the simulated noise of 25 dB, which is a signal-to-noise ratio found in very good quality real ERP dataset. All simulated recordings used the LFP modality of DCM code and the pyramidal cell activity of each region was observed with unitary gain. With this configuration, the variational Bayes optimization scheme directly fits the local neuronal dynamics to infer the parameters of the model, instead of using a lead-field weighted linear combination of all neuronal activities supposed to generate EEG or MEG signals.

#### Fit procedure

Each dataset was fitted with 3 models: two *false* models based on the structural architecture of the generative model but allowing respectively for modulations of forward connections only (F) and backward connections only (B), and the *true* generative model allowing for modulations of both forward and backward connections (FB). All models were equipped with DDE_TA integration scheme and had the same default priors. In particular, synaptic time constants and conduction delays had prior expectations as mentioned in Section 2.1 and the same relatively low prior precision (DCM standard value of 16). This ensured that all models were offered the same freedom to move these parameters away from their prior values.

#### Bayesian model comparison and parameter estimates

A random effect (RFX) Bayesian model comparison (BMC) was used to compute the expected posterior probability for each of the 3 models (Penny et al., 2010, Stephan et al., 2009). RFX was preferred to fixed effect (FFX) modeling because it frees the inference from the assumption that datasets were generated with a common model. Having identified the model with highest expected posterior probability, a mean posterior density on modulation parameters was estimated by averaging the posterior distribution estimated for each dataset weighted by the posterior probability of the model for this dataset. Finally, these parameter estimates were compared to the true generative values. The full estimation procedure was then repeated with models equipped with DDE_RK and parameter estimates were compared to those obtained with DDE_TA. The reason for this is not to compare the integration schemes, which would be a tautology in this context, but rather to separate the inaccuracies in the parameter estimation due to the approximation done by DDE_TA from those due to the optimization scheme. In this sense, estimates obtained with DDE_RK were considered *a priori* as a reference of what could be obtained without any approximation.

#### Simulation 1: model with 2 regions

**Fig. 1:**
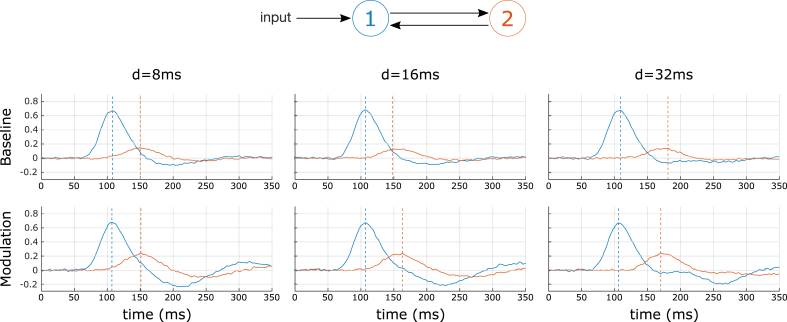
Data used for the first simulation. Top: ERP DCM model composed of 2 regions connected with forward and backward connections. Bottom: Activities of the 2 regions generated by the model equipped with DDE_RK. Each dataset comprises a baseline (first row) and a modulated (second row) conditions. The modulation affects both forward (F) and backward (B) connections. Activities are presented for 3 values of the conduction delay d (in the 3 columns): 8 ms, 16 ms and 32 ms. Dotted lines indicate the peak latency in each region.

In this simulation, the generative model was composed of 2 regions reciprocally connected with forward and backward connections and a stimulus input entering region 1 (Fig. 1). In the modulated condition of each dataset, forward and backward connections were modulated by a factor of 0.5 and 0.8 respectively compared to the baseline condition. These strengths of modulation^1^ were chosen in order to get a notable effect of the modulation while keeping the system stable. We generated a series of 10 datasets with an SNR of 25 dB for every symmetrical conduction delay between regions 1 and 2 (from 4 ms up to 32 ms with a step of 4 ms). Typical generated time courses are shown in Fig. 1; they illustrate clearly the non-linear interaction between conduction delays and the effects of the modulation.

#### Simulation 2: model with 6 regions

**Fig. 2:**
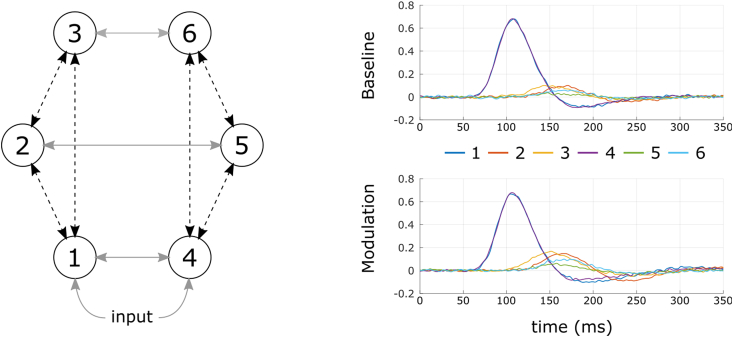
Model and example dataset used for the second simulation. Left: The generative model was composed of 6 regions. All forward and backward connections (dotted black lines) were modulated while lateral and input connections (solid gray lines) were kept constant. Right: Example of a dataset generated for the baseline (top) and the modulated conditions (bottom). Strength of connection and conduction delays were random parameters while the modulation was fixed to 0.5 for all forward and backward connections (SNR = 25 dB).

In this simulation, the generative model was composed of 6 regions reciprocally connected with forward, backward and lateral connections and a stimulus input entering regions 1 and 4 (Fig. 2). For each dataset, all connection strengths were uniformly sampled from the interval [0 2]^2^ while conduction delays between reciprocally connected regions were uniformly sampled between 8 ms and 32 ms. In the modulated condition, all forward and backward connections were modulated with a strength of 0.5. A series of 100 datasets was generated with an SNR of 25 dB. Typical time courses are presented in Fig. 2. Due to the number of regions interacting in this model and the full variability of connection strengths and conduction delays used to generate the data, this simulation reproduced a more realistic and non-trivial situation.

### Application: DCM of mismatch negativity EEG dataset

We compared the two integration schemes while fitting an ERP dataset from a subject recorded with 128 EEG channels during an auditory mismatch negativity (MMN) paradigm (dataset publicly available at www.fil.ion.ucl.ac.uk/spm/data/eeg_mmn). In this experiment, standard (500 Hz) and deviant (550 Hz) tones were randomly presented to the subject with respective frequencies of occurrence of 80% and 20%. The MMN effect refers to the difference between the evoked responses to standard and deviant tones; it corresponds to a negative component present only in the deviant condition. In this dataset, the baseline condition is the average response to standard tones. The other condition is the average response to deviant tones and is considered as the modulated condition.

**Fig. 3:**
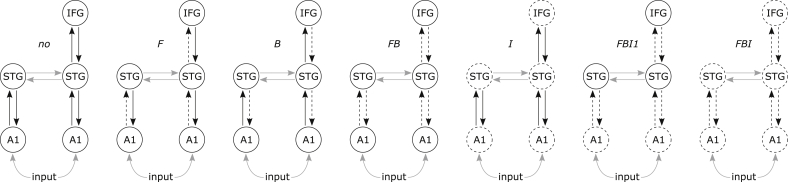
Set of dynamic causal models (DCMs) used to fit the MMN dataset. All models have the same structure composed of 5 regions (bilateral A1: primary auditory cortices; bilateral STG: superior temporal gyri; right IFG: inferior frontal gyrus) reciprocally connected with forward/backward (black lines) and lateral (gray lines) connections. The models differ by the connections being modulated (dashed black lines) in the deviant tone condition. Stimulus input enters each model bilaterally in A1.

Using DCM for ERP, different hypotheses have been formulated in terms of modulations of effective connectivity between the brain regions engaged in the processing of the stimuli, in order to account for the MMN effect (Garrido et al., 2007). In the present analysis, we focused on a subset of 7 models reflecting different mechanistic hypotheses (Fig. 3). All models were based on the same anatomical architecture but differed by the connections allowed to be modulated between the two conditions of interest. The first model (no) did not allow for any modulation (null model, which served as a reference). Following models allowed for only forward (F), only backward (B) or both forward and backward (FB) connections to be modulated. The model I allowed for only intrinsic modulations of all regions. The last models allowed for both forward and backward modulations in addition with either only 1st level primary auditory regions (FBI1) or all regions (FBI) intrinsic modulations. The ERP dataset was fitted with these models and the estimation procedure was repeated separately for each integration scheme (DDE_TA and DDE_RK).

## Results

### Integration of a simple forward model with 2 regions

**Fig. 4:**
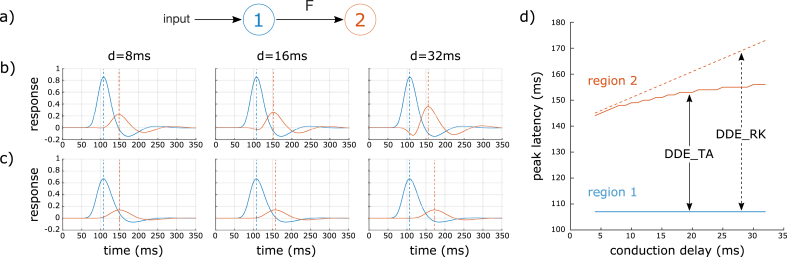
Examples of data generated by DDE_TA and DDE_RK integration schemes. a) The DCM model composed of regions 1 (blue) and 2 (red) connected with a forward (F) connection. Responses are generated for conduction delays of 8 ms, 16 ms, and 32 ms between the 2 regions, with either DDE_TA (b) or DDE_RK (c) integration schemes. Dotted line identifies the peak latency for each region. d) Latency of peak response for the 2 regions for different conduction delays.

We first generated data from a simple ERP neuronal model comprising 2 regions connected with a forward connection and varied the extrinsic conduction delay between region 1 and 2 from 4 ms to 32 ms (Fig. 4). Using DDE_RK, increasing conduction delay has the only effect to shift the response of region 2 by the same amount of time, which is the expected behavior for this model. Using DDE_TA, it produces unexpected modulation effects on the response of region 2: the apparition of an early negative response between 90 ms and 120 ms can be noted, as well as an increase of the peak amplitude and an attenuation of the shift of the peak latency of region 2. There is also systematic higher amplitude of the initial response in region 1 (around 100 ms) with DDE_TA compared to DDE_RK; this may be due to the different step-size strategy used during integration. The over sparse estimation of the dynamics $\dot{x}\left(t\right)$ by DDE_TA results in an imprecise estimation of this first response. It is clear from this data generation result that the assumptions made by DDE_TA are no longer valid for propagation delays higher than 5–10 ms in this simplistic simulation.

### Simulation 1: parameter estimation and model comparison using the model with 2 regions

**Fig. 5:**
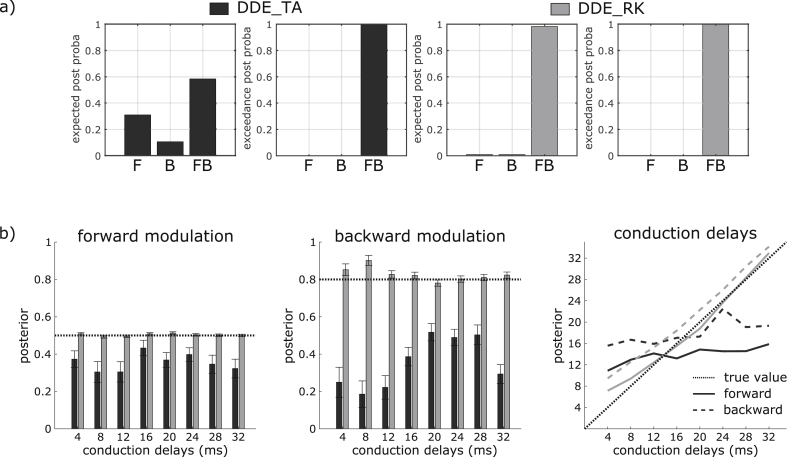
Summary of results from Simulation 1. a) RFX Bayesian model comparison between the three models: F, B, and FB, for the 2 integration schemes, DDE_TA (two first plots, in black) and DDE_RK (two next plots, in gray). The true generative FB model is identified with an exceedance posterior probability p = 0.99 in both cases. b) Posteriors estimation using DDE_TA (in black) and DDE_RK (in gray): Forward (left plot) and backward (middle plot) modulations are represented with their 90% credibility interval; the dotted horizontal line corresponds to the true generative parameter in each plot. Rightmost plot: Posterior conduction delays obtained with DDE_TA (in black) and DDE_RK (in gray) are represented for forward (plain line) and backward (dashed line) connections; the dotted black line represents the true conduction delay values.

Using the data generated with the FB model equipped with DDE_RK presented on Fig. 1, we proceeded to the Bayesian model comparison among F, B, and FB models across all conduction delays (Fig. 5a, left). The FB model integrated with DDE_TA was more plausible than the F and B models, with an expected posterior probability of p = 0.58 and with a high confidence (exceedance probability, p = 0.99). This means that, for any randomly selected dataset, there was an important posterior probability (total expected posterior probability, p = 0.31 + 0.11 = 0.42) for this data to have been generated by either the F or the B model and therefore, that one modulation present in the data (backward or forward, respectively) was not significantly detected. The presence of these false negatives indicates either that the absent modulation is compensated by other parameters of the model or that the potential improvement in accuracy of the FB model is not sufficient to counterbalance its additional complexity. In contrast, considering the same models integrated with DDE_RK, the FB model was clearly identified, with an expected posterior probability of p = 0.98 (Fig. 5a, right).

For each conduction delay, average posteriors were computed from the corresponding series of 10 datasets fitted with the winning FB model. Fig. 5b shows the posterior parameter estimates (mean, 90% credibility interval, and true parameter) for forward and backward modulations. The systematic underestimation of forward and backward modulations, previously mentioned for F and B models, was also found with the FB model. It may be due to the bias in amplitude and shape introduced into the responses of the regions by the use of both a non-adaptive step size and the Taylor approximation during the integration of the dynamical system (Fig. 4b). A second observation is that the Taylor approximation (with respect to conduction delays) did not provide better estimates even at shorter delays. This was partly due to the inaccurate estimation of the conduction delays (Fig. 5b, right), which hardly moved away from their prior values (16 ms) despite a low prior precision. Indeed, DCM scheme using DDE_RK revealed that the same prior precision allowed for the drift of parameters and provided accurate estimates of conduction delays, and in particular when the true conduction delays of 32 ms were far away from the prior values of 16 ms. However, there was a slight but systematic overestimation of the backward conduction delays, ranging from 6 ms for shortest delays to 2 ms for largest delays.

### Simulation 2: parameter estimation and model comparison using the model with 6 regions

**Fig. 6:**
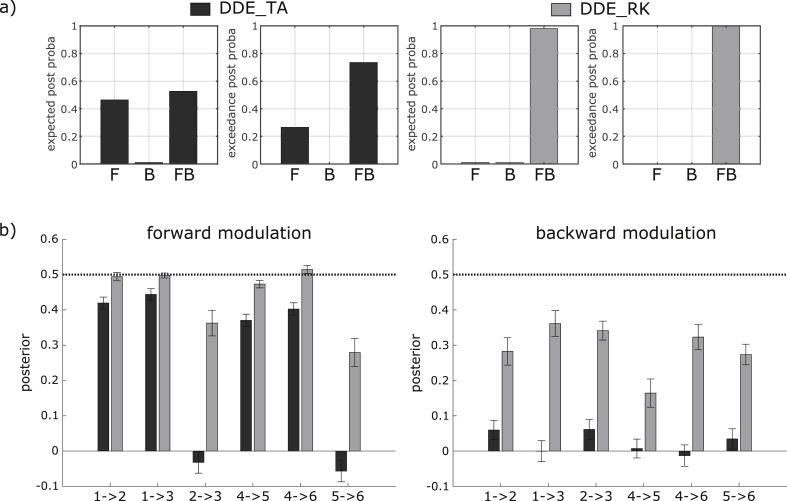
Summary of results from Simulation 2. a) RFX Bayesian model comparison between the three models: F, B and F, for the 2 integration schemes, DDE_TA (two first plots, in black) and DDE_RK (two next plots, in gray). The true FB model was identified with good confidence with DDE_TA (exceedance probability, p = 0.73) and DDE_RK (exceedance probability, p = 1.00). b) Posteriors estimation of forward (left plot) and backward (right plot) modulations using DDE_TA (in black) and DDE_RK (in gray), represented for each connection with their 90% credibility interval. The dotted, black, horizontal line corresponds to the true generative parameters in each plot.

Bayesian model comparison among F, B, and FB models fitted to the series of 100 datasets generated from the 6 source models is presented in Fig. 6a, left. Using DDE_TA, the posterior probability was estimated higher for the generative FB model (p = 0.53) than for the F model (p = 0.46) and the B model (p = 0.01). Despite the rather small difference between the FB and the F models, the FB model was estimated more plausible than the F model with good confidence (exceedance probability, p = 0.73). In contrast, Bayesian comparison of models fitted using DDE_RK (Fig. 6a, right) only pointed out the FB model, with expected posterior probability of p = 0.98 and exceedance probability of p = 1.00.

The average posteriors of modulation (and their 90% credibility interval) are shown in Fig. 6b, separately for each forward and each backward connections. With DDE_TA, the estimation of the modulation for the forward connections 1->2, 1->3, 4->5 and 4->6 were rather close to the true value, whereas the forward connections 2->3, 5->6 and all backward modulations were very poorly estimated. In contrast, with DDE_RK, even if backward modulations were not perfectly retrieved (thus showing a similar overall tendency as with DDE_TA), forward modulations were estimated with high precision (in particular for connections 1->2, 1->3 and 4->6 where the true modulation lied in the tight 90% credibility interval).

The estimates obtained by models equipped with DDE_RK were clearly the most accurate, yet the backward connections were underestimated. We interpret this result as a weak effect of the modulation of backward connections on the generated data. Indeed, because of random strengths of connection used to generate the datasets, some regions had very weak responses (in both the baseline and the modulated conditions), in which the effect of the modulation could easily be hidden by noise. If we consider only interactions among regions 1, 2, and 3, the first effects of modulation appeared around 120 ms in the responses of regions 2 and 3 (Fig. 2) and corresponded to the modulation of connections 1->2 and 1->3, which were estimated accurately. Due to synaptic and conduction delays, the following main effects of modulation started from 170 ms for the other forward (2->3) and backward connections (2->1, 3->1 and 3->2) and were estimated with less accuracy. These late components (and associated modulations), which were usually subtler than the early ones in our generated datasets, were therefore harder to detect for the optimization procedure. The associated decrease in precision also reflects a (temporo-spatial) propagation of parameter estimation errors, which is accentuated when using the DDE_TA integration scheme.

Overall, the results of the second simulation confirmed those of the first simulation in a more general context. The precision of the integration scheme had effects on the Bayesian model comparison (with less clear identification of the generative model) and on the estimation of connectivity parameter posteriors (with a loss of precision, in particular concerning late components).

### Application: Mismatch negativity EEG dataset

**Fig. 7:**
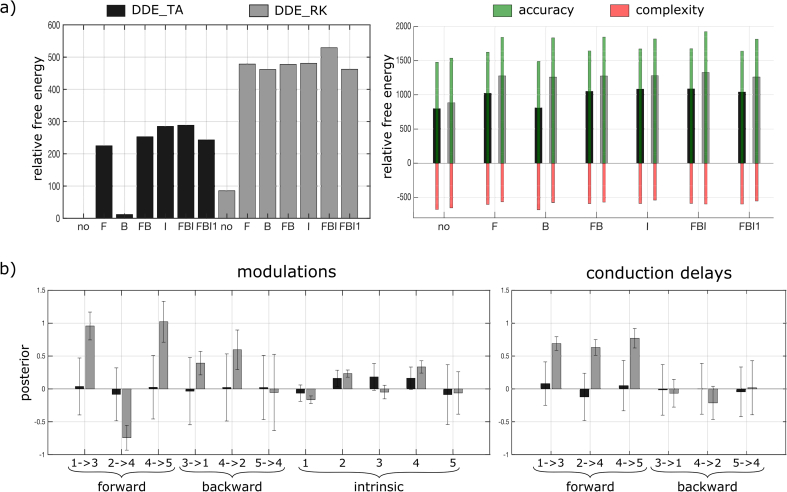
DCM results on a MMN dataset. a) Left: Comparison of the relative free energy between the 7 tested models for DDE_TA (in black) and DDE_RK (in gray). Right: Decomposition of the relative free energy into accuracy (green) and complexity (red). b) Posterior estimation of forward, backward and intrinsic modulations (left plot) and conduction delays (right plot) with their 90% credibility interval for the winning model FBI integrated with either DDE_TA (in black) or DDE_RK (in gray) schemes.

Bayesian model comparison in terms of relative free energy is shown in Fig. 7a (left) for the seven models and the two integration schemes tested on the publicly available MMN dataset from an example subject. The best model was FBI integrated with DDE_RK (posterior probability, p = 1.0). In addition, each model integrated with DDE_RK had higher evidence than the same model integrated with DDE_TA. Even if the BMC restricted to models using DDE_TA also selected FBI as the best model with very high confidence (posterior probability, p = 0.98), the relative ranking of other models was different (Fig. 7a, left). The ranking of the models (and their respective relative free energy) using DDE_TA was: 1: FBI (289), 2: I (285), 3: FB (253), 4: FBI1 (243), 5: F (225), 6: B (12), 7: no (0). The ranking of the models using DDE_RK was: 1: FBI (529), 2: I (481), 3: F (479), 4: FB (478), 5: FBI1 (462), 6: B (462), 7: no (86).

Moreover, the free energy (or log evidence) of a model can be decomposed into an accuracy term and a penalizing complexity term^3^ (Fig. 7a, right). This distinction showed that the systematic difference in free energy, for each model, in favor of DDE_RK relative to DDE_TA, was mostly due to a better accuracy.

For the winning FBI model, estimation of posterior means (and 90% credibility intervals) for modulations and conduction delays was compared between the two integration schemes (Fig. 7b). For each parameter, the tendencies of variation were relatively similar (an increase or a decrease relatively to the prior zero value) but the amplitudes of variation were much more pronounced with DDE_RK. Although the true generative parameters for this dataset are unknown, the present results suggest the same under-estimation of modulation and the same imprecise estimation of conduction delays as observed in the simulations (Fig. 6, Fig. 5b).

## Computational considerations

The gain in precision has a cost. When the dynamics of the system is fast, the *dde23* algorithm, used in this note to implement DDE_RK, makes use of very small time steps to ensure the expected precision. Added to the fact that at each time step, the resolution of the system for various time-delay instants is needed, solving such a system of DDEs induces a significant cost in computational time (which increases exponentially with the complexity of the model). For instance, fitting the 7 models to the MMN dataset with DDE_TA took approximately 5 min per model while the same operation needed 6 h per model with DDE_RK (see Supplemental data). In return, this integration scheme appeared to be extremely stable across different operating systems. Optimized successively on macOS and Linux systems, the free energy was estimated with a mean absolute difference (across models) of 15.96 for DDE_TA but only 0.41 for DDE_RK.

All the results above were computed using either DDE_TA with a fixed step size (data sampling rate: 1 ms for the simulations and 5 ms for the MMN dataset) or DDE_RK with the default MATLAB options (0.1% for the relative error tolerance and 1e-6 for the absolute error tolerance). To look for a better compromise between the precision of the estimates and the increase in computational load, the parameterization of the integration schemes were further investigated: DDE_TA with a smaller step size and DDE_RK with a higher error tolerance (Table 1 show a summary of the results, see Supplementary data for the full details on this analysis). Using a smaller step size of 0.1 ms for DDE_TA had no effect on the accuracy of the integration and was therefore not considered further. However, increasing the relative error tolerance of DDE_RK up to 1% appeared to be a good way to save computational time, at the cost of a limited inaccuracy to retrieve the generative parameters of the simulations. Interestingly, all the models used to fit the (simulated or real) data systematically obtained higher evidence with DDE_RK (with either a relative error tolerance of 0.1% or 1%) compared to DDE_TA. For the MMN dataset, a higher relative error tolerance of 1% (instead of the default more stringent 0.1%) controlled by the DDE_RK integration scheme saved 52% of computational time.

**Table 1:** Median computational time for the simulations and the MMN dataset, using the integration schemes DDE_TA and DDE_RK with a relative error tolerance (RelTol) of 0.1% (default) and 1%. See Supplementary Data for an analysis of DDE_RK relative error tolerance.

	*DDE_TA*	*DDE_RK, RelTol*=*0.1%*	*DDE_RK, RelTol*=*1%*
Simulation 1	1mn56s	30mn33s	18mn55s
Simulation 2	16mn53s	14h20mn	12h36mn
MMN dataset	4mn30s	6h05mn	2h56mn

## Discussion

In this note, we first showed that differences between integration schemes have significant effects on modeled responses when varying conduction delays, such as modulation of the shape of the responses and attenuation of the expected peak latency offset with increasing conduction delays. Second, simulations showed that, given sufficient amount of data with a high SNR, the BMC procedure using DDE_TA retrieved the generative model with high confidence, but still assigned a lower posterior probability to the model, compared to DDE_RK. Interestingly, the first simulation showed that the loss of precision in the estimation of the modulation was independent of the conduction delay. The second simulation replicated this result in a more challenging and realistic configuration using a model composed of 6 sources. The variability one can expect in DCM group studies was introduced using connections with random strengths. The effects of the modulation were non-uniformly assigned to the connections through all the datasets (a weak connection carry almost no effect of modulation) and Bayesian procedures had to gather the information available (and unequally spread) in each dataset to properly infer the generative parameters at the group level. BMS identified well the FB model as being more plausible than either the F or the B models, with high confidence (exceedance probability, p = 0.73). However, the late effects of backward modulations were not as well recovered as earlier effects of forward modulations. It could reflect that in this simulation, the late components had smaller amplitude than the early ones, relative to a same level of noise and thus exhibited more subtle effects of the modulation which were harder to detect, especially using DDE_TA. Finally, fitting seven DCM models to a MMN dataset revealed, for any model architecture taken separately, the higher goodness of fit obtained by DDE_RK compared to DDE_TA, along with a stronger deviation of the parameters (in posteriors estimates) from their prior distribution (especially for forward and backward modulations), suggesting a larger degree of freedom of the system dynamics. Assessing the complete added-value of Runge-Kutta methods for experimental data is difficult without crossed validating measures, as the ground truth remains unknown. However, by fitting a whole series of real datasets with DDE_TA and DDE_RK and comparing their relative free energy (and its decomposition into accuracy and complexity), it would be possible to at least validate the increased goodness of fit provided by DDE_RK.

The initial objective of the present study was to assess the effects of the 1st order Taylor approximation used to integrate the biophysical equations of DCM for ERPs. Briefly, with this approximation, the simulations showed that 1) the Bayesian model selection was robust enough to identify the generative model; 2) the estimation of parameters were relatively poorly estimated as compared to when a Runge-Kutta method was used. These findings were clear from simulations, and the use of a real MMN dataset gave some hints that it might also be true for experimental data. However, due to the important additional computation load introduced by Runge-Kutta methods, it is not realistic to apply them blindly, especially for large DCM group studies inverting many models. Given the present results, if DCM analysis cannot be practically carried out fully with DDE_RK, we suggest to proceed to the Bayesian model selection using all models inverted with DDE_TA. Then, for parameter inference, only winning models could be inverted again, this time using DDE_RK to provide a better parameter estimation. Another interesting option could be, when possible, to use Bayesian model reduction (Friston et al., 2015). This technique “provides an efficient way to invert large numbers of (reduced) models, following the (usually computationally expensive) inversion of a full model”. Indeed, “the posterior of a reduced model can be derived from the posterior of the full model”. In this approach, DDE_RK would be used only once to fit the full parent model, resulting in a considerable time-saving.

Overall, our results suggest that in most cases the DCM for ERP methodology should benefit a lot from the use of the Runge-Kutta integration scheme. While the implemented integration scheme based on 1st order Taylor approximation with fixed step size represents a powerful compromise between accuracy and computation time, it becomes inappropriate when the conduction delays are of significant importance for the dynamics of the system. This may be not the case for some configurations, *e.g.* the study of small local systems, but experimentally latencies of 20 ms and more have been reported in human brain networks, *e.g.* from cortico-cortical evoked potentials triggered by focal direct stimulations (David et al., 2013). In addition, relying on an accurate integration of the system of DDEs key to DCM models is particularly important when neural sources are likely to show fast ERP dynamics, *e.g.* early phasic responses from primary sensory cortices.

## Software note

The MATLAB (R2016b) code used in this technical note is available for download at f-tract.eu/publications. Built on SPM (r6732), it contains the ready-to-use implementation of the DDE_RK integration scheme and the procedure to reproduce the results of the present study.

## Conflicts of interest

The authors declare no conflict of interest.

## Footnotes

1 These are log-scale values; this means that for forward modulation, if *c* is the strength of 1->2 connection in the baseline condition, then the strength of 1->2 connection in the modulated condition is c*exp(0.5) = c*1.65, reflecting higher forward connectivity between regions 1 and 2.

2 These are not log-scale values: a null connection strength corresponds to absent connection.

3 In the DCM code (see spm_dcm_erp.m and spm_nlsi_N.m), these quantities are accessible for each fit within the array variable DCM.L. The sum of terms 1, 6 and 7 (resp. 2, 3, 4, 5, 8 and 9) corresponds to the accuracy (resp. to the negative complexity) of a fit. The sum of all the terms is the variational free energy of the fit. This ensues from the mathematical description given in Eq. (18) and Eq. (19) of (Penny et al., 2004).

Appendix A Supplementary data related to this article can be found at https://doi.org/10.1016/j.neuroimage.2018.02.031.
