# Supplementary material for: Comparison of two integration methods for dynamic causal modeling of electrophysiological data
Source: Neuroimage. 2018 Jun;173:623–31. doi: 10.1016/j.neuroimage.2018.02.031 (PMC5929904; doi:10.1016/j.neuroimage.2018.02.031)
Supplement: Appendix [file mmc1.docx]

# Appendix

## Use of DDE_RK integrator for DCM of ERP

In the current implementation of DCM (SPM r6732), the default integration scheme is DDE_TA. To switch to a DDE_RK integration scheme, it has to be specified in the model definition as follows:

% switch from DDE_TA to DDE_RK

DCM.M.IS = 'spm_gen_erp_dde23' ;

% set relative and absolute error tolerance options for dde23

DCM.M.dde23_options = struct( 'reltol', 1e-3, 'abstol', 1e-6 ) ;

% run the fit procedure

spm_dcm_erp( DCM ) ;

## Implementation of DDE_RK integrator for DCM of ERP

In the current implementation of DCM (SPM r6732), the integration of the system of DDEs described in Eq. (1) is implemented in the routine:

function [y,pst] = spm_gen_erp(P,M,U)

This routine uses DDE_TA integration scheme to generate the ERP for the parameters P of a given neuronal model M, in response to a stimulus input U. To implement the DDE_RK integration scheme for DCM, we provide the equivalent routine:

function [y,pst] = spm_gen_erp_dde23(P,M,U)

This routine uses the dedicated MATLAB (R2016b) DDEs solver dde23 to integrate the system of DDEs Eq. (1):

sol = dde23(ddefun, lags, history, tspan, options)

The meaning of these arguments in the current context of DCM is as follows:

sol the global state of the system: **x**

ddefun the function describing the dynamics of the system: $\boldsymbol{\varphi}_{\boldsymbol{\theta}}$

lags the (extrinsic and intrinsic) conduction delays: $d_{kl}$

history the pre-stimulus state of the system (equilibrium)

tspan the time window of integration (duration of the ERP)

options the solver properties (control of the error tolerance)

Because the routine dde23 uses an adaptive step size, the result sol is finally resampled at the sampling rate of the ERP.
