# Supplementary material for: Comparison of two integration methods for dynamic causal modeling of electrophysiological data
Source: Neuroimage. 2018 Jun;173:623–31. doi: 10.1016/j.neuroimage.2018.02.031 (PMC5929904; doi:10.1016/j.neuroimage.2018.02.031)
Supplement: Supplementary data [file mmc2.docx]

**Supplementary** **data**

**Comparison of two integration methods for dynamic causal modeling of electrophysiological data**

Jean-Didier Lemaréchal^1,2,3,4^, Nathalie George^1,2^, Olivier David^3,4*^

*^1^ Sorbonne Universités, UPMC Univ Paris 06, Inserm, CNRS, Institut du Cerveau et de la Moelle épinière (ICM), - Hôpital Pitié-Salpêtrière, Boulevard de l’hôpital, F-75013, Paris, France*

*^2^ Sorbonne Universités, UPMC Univ Paris 06 UMR S 1127, Inserm U 1127, CNRS UMR 7225, Institut du Cerveau et de la Moelle épinière, ICM, Ecole Normale Supérieure, ENS, Centre MEG-EEG, F-75013, Paris, France*

*^3^ Inserm, U1216, F-38000, Grenoble, France*

*^4^ Univ. Grenoble Alpes, Grenoble Institut des Neurosciences, GIN, F-38000 Grenoble, France*

# Introduction

In the main text, all the computation used DDE_TA with a fixed step size (data sampling rate: 1ms for the simulations and 5ms for the MMN dataset) and DDE_RK with the default MATLAB options (0.1% for the relative error tolerance and 1e-6 for the absolute error tolerance). Here, we consider the possibility to find a compromise between computational efficiency and accuracy for the two integration schemes. Therefore, we further investigate the influences of:

- a smaller step size for DDE_TA
- a higher error tolerance for DDE_RK

For this purpose, we revisited each part of the Results section. The computation time (CT) and the estimation error are presented, when it is appropriate.

# Integration of a simple forward model with 2 regions

Here, the model presented on Fig. 4 in the main text is used to generate the data.

First, for DDE_TA, step sizes of 1ms and 0.1ms are compared and the generated data are presented, superimposed, on Fig. 1a. Decreasing the step size has almost no visible effect (for all delays considered) on the unexpected modulations of the responses and the attenuated shift of the peak latency. It only linearly increases the computation time (CT=0.8s for 1ms and CT=7s for 0.1ms).

Second, for DDE_RK, a relative error (RelTol) tolerance of 0.1% is compared to 1% and the generated data are presented, superimposed, on Fig. 1b. This produces no significant difference between the waveforms but saves 33% of CT (CT=2.1s for RelTol=0.1% and CT=1.4s for RelTol=1%). Indeed, increasing the error tolerance allows DDE_RK to use larger steps during the integration (the minimum step size is 0.7ms for RelTol=0.1% and 1ms for RelTol=1%) while keeping its adaptive behavior (see Fig. 1c and d). Controlling the relative error tolerance (instead of the absolute error tolerance) was suited to DCM for ERPs because responses with high or low amplitude are as much likely to show the effects of modulation and therefore request a comparable precision.

Considering these results, DDE_TA using a smaller step size was abandoned and we now focused only on DDE_RK with RelTol=1% to evaluate its ability to recover precise parameter estimates.

| 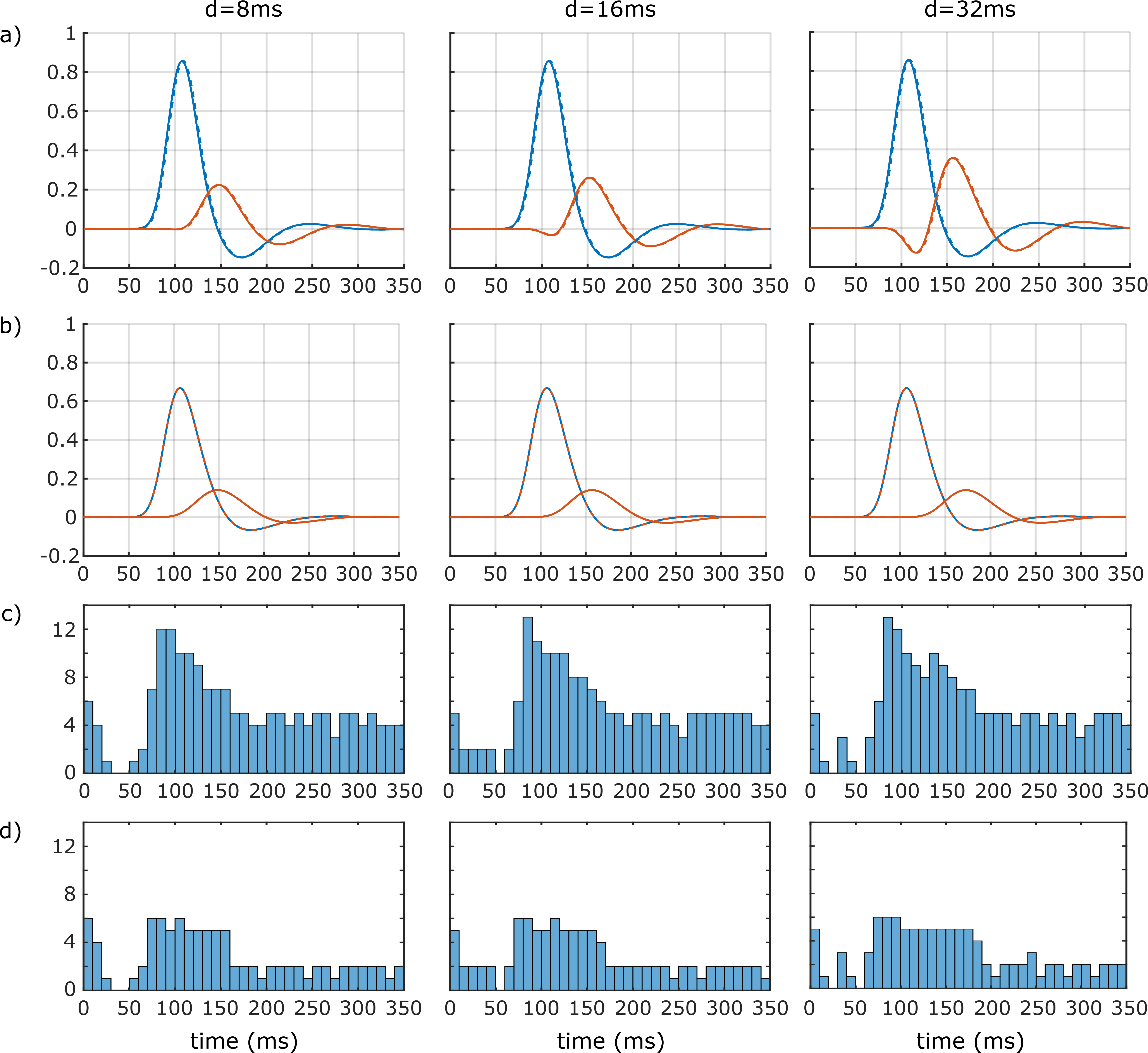 |
| --- |
| Fig. 1 Data generated from the DCM model composed of regions 1 (blue) and 2 (red) connected with a forward (F) connection (see Fig. 4, main text) and conduction delays of 8ms, 16ms, and 32ms. a) Superimposition of the responses using DDE_TA with a step size of 1ms (plain line) and 0.1ms (dashed line) and b) using DDE_RK with a relative error tolerance (RelTol) of 0.1% (plain line) and 1% (dashed line). c) Repartition of the adaptive steps used during integration by DDE_RK with RelTol=0.1% and d) RelTol=1%. |

# Evaluation of DDE_RK with a relative error tolerance of 1%

The procedures used in the simulations and the fit of the MMN dataset were repeated, using the integration scheme DDE_RK with a relative error tolerance of 1% to fit the data. The results presented Fig. 5, 6 and 7 in the main text were extended accordingly and are presented in the following sections. For a precise comparison between the three integration schemes, parameter estimation error and computation time are also reported.

## Simulation 1: parameter estimation using the model with 2 regions

The Bayesian model comparison for DDE_RK, RelTol=1% identified the true generative model FB across all conduction delays with an expected posterior probability of p=0.89 (p=0.58 for DDE_TA and p=0.98 for DDE_RK, RelTol=0.1%).

| 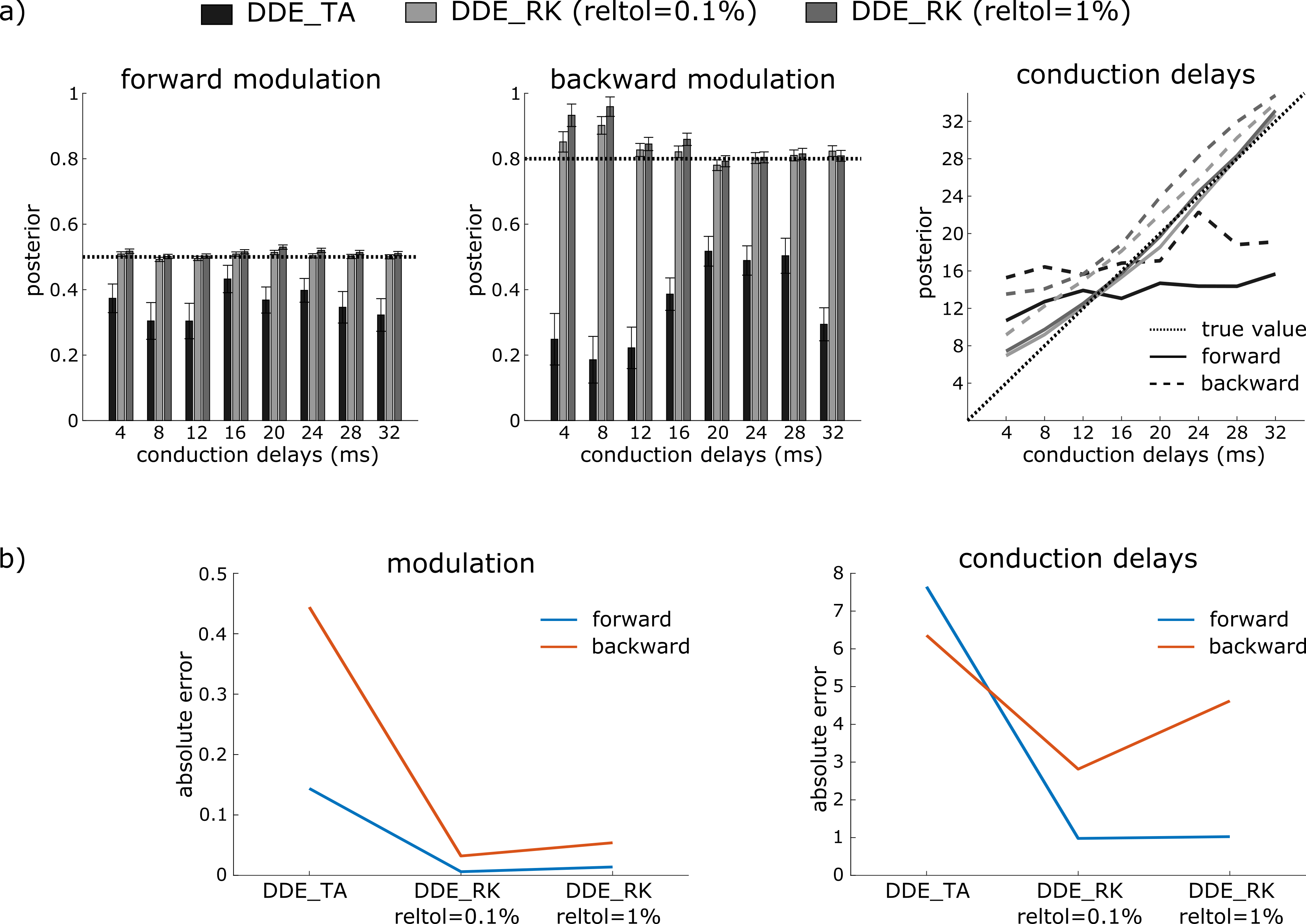 |
| --- |
| Fig. 2 a) Posteriors estimates from simulation 1 using DDE_TA (in black), DDE_RK, RelTol=0.1% (in light gray) and DDE_RK, RelTol=1% (in dark gray): Forward (left plot) and backward (middle plot) modulations are represented with their 90% credibility interval; the dotted horizontal line corresponds to the true generative parameter in each plot. Rightmost plot: Posterior conduction delays for each integration scheme are represented for forward (plain line) and backward (dashed line) connections; the dotted black line represents the true conduction delay values. b) Mean absolute estimation error (across conduction delays) for forward and backward modulation (left) and conduction delays (right). |

The posterior of forward and backward modulations estimated with DDE_RK, RelTol=1% are slightly less accurate than those obtained with DDE_RK, RelTol=0.1% but still very close from the true generative values (see Fig. 2 and Table 1a).

Forward conduction delays are also estimated very precisely (absolute error of 1ms), given the sampling rate of the data (1000 Hz). All backward conduction delays are clearly overestimated (mean absolute error of 4.6 ms) and this is particularly emphasized for conduction delays inferior to 12ms.

Importantly, the use of DDE_RK, RelTol=1% saves 38% of computational time compared to DDE_RK, 0.1%(see Table 1b).

| *a) Mean absolute (relative %) error* | | | |
| --- | --- | --- | --- |
|  | *DDE_TA* | *DDE_RK, RelTol=0.1%* | *DDE_RK, RelTol=1%* |
| *F modulation* | 0.14 (28.8%) | 0.01 (1.2%) | **0.01 (2.8%)** |
| *B modulation* | 0.44 (55.5%) | 0.03 (4.0%) | **0.05 (6.7%)** |
| *F cond. delay* | 7.6 ms (53.4%) | 1.0 ms (13.3%) | 1.0 ms (15.2%) |
| *B cond. delay* | 6.4 ms (64.7%) | 2.8 ms (31.5%) | 4.6 ms (52.8%) |
|  | | | |
| *b) Computation time* | | | |
|  | *DDE_TA* | *DDE_RK, RelTol=0.1%* | *DDE_RK, RelTol=1%* |
| *Median* | 1mn56s | 30mn33s | **18mn55s** |
| *Minimum* | 55s | 18mn5s | 13mn52s |
| *Maximum* | 6mn25s | 1h56mn | 36mn38s |
|  |  |  |  |
| Table 1 Comparison of the performances of the three integration schemes for simulation 1. a) Mean absolute (and relative) error for estimates of forward and backward modulation and conduction delays. b) Computation time obtained during the fit. | | | |

## Simulation 2: parameter estimation using the model with 6 regions

The Bayesian model comparison for DDE_RK, RelTol=1% pointed out the true generative model FB across all conduction delays with an expected posterior probability of p=0.98 (p=0.53 for DDE_TA and p=0.98 for DDE_RK, RelTol=0.1%).

| 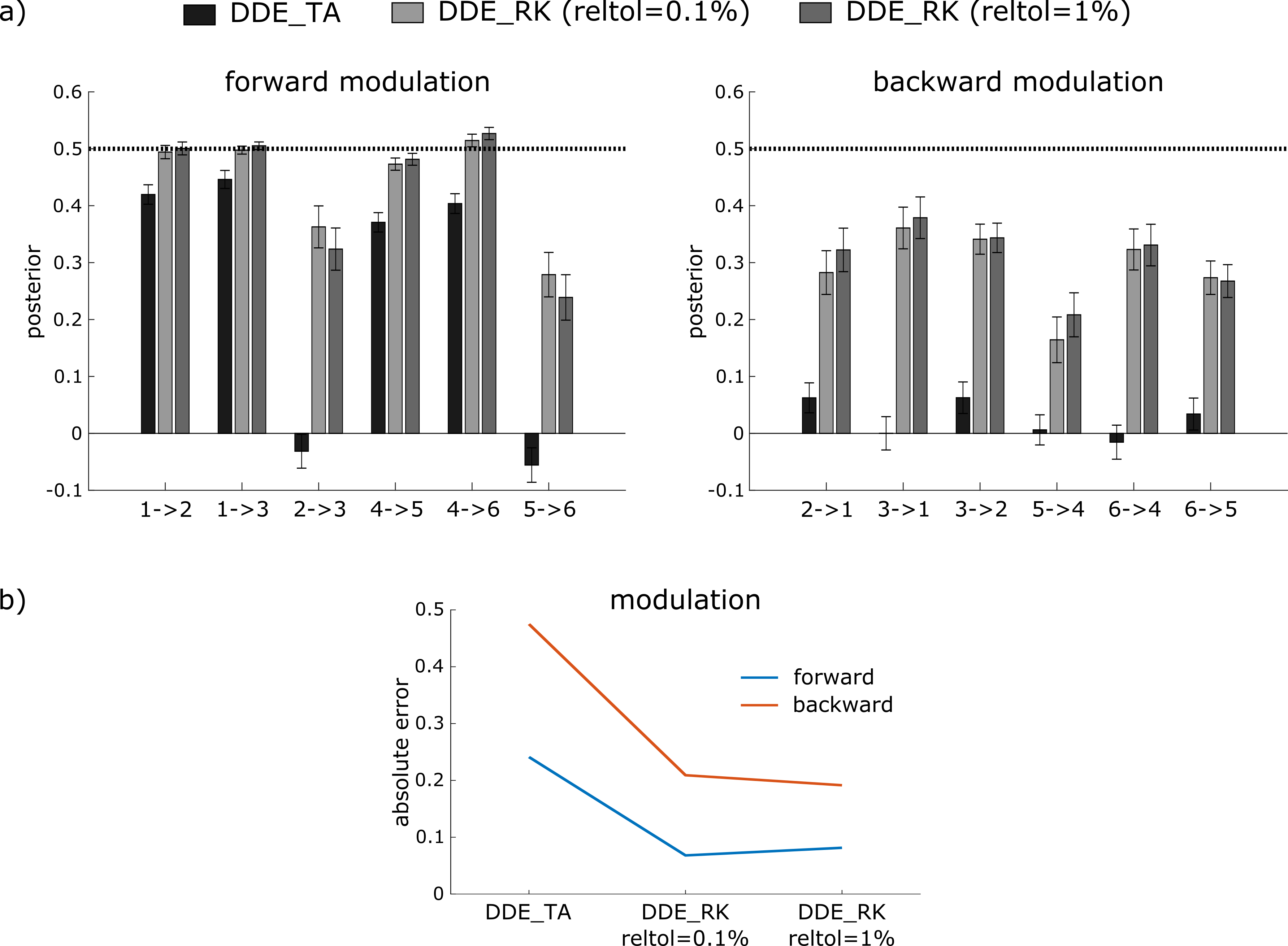 |
| --- |
| Fig. 3 a) Posteriors estimates from simulation 2 using DDE_TA (in black), DDE_RK, RelTol=0.1% (in light gray) and DDE_RK, RelTol=1% (in dark gray): Forward (left plot) and backward (right plot) modulations are represented with their 90% credibility interval; the dotted horizontal black line corresponds to the true generative parameter in each plot. b) Mean absolute estimation error for forward and backward modulation for each of the three integration schemes. |

In this simulation, the estimation of forward and backward modulation with DDE_RK, RelTol=1% and DDE_RK, RelTol=0.1% were very close to each other (see Fig. 3a). An expected slight decrease (2.7% of mean relative error) of precision was observed for the forward modulation while a surprisingly moderate increase (3.5% of mean relative error) of precision was observed for the backward modulation (see Fig. 3b and Table 2a).

| *a) Mean absolute (relative %) error* | | | |
| --- | --- | --- | --- |
|  | *DDE_TA* | *DDE_RK, RelTol=0.1%* | *DDE_RK, RelTol=1%* |
| *F modulation* | 0.24 (48.2%) | 0.07 (13.6%) | **0.08 (16.3%)** |
| *B modulation* | 0.48 (95.0%) | 0.21 (41.8%) | **0.19 (38.3%)** |
|  | | | |
| *b) Computation time* | | | |
|  | *DDE_TA* | *DDE_RK, RelTol=0.1%* | *DDE_RK, RelTol=1%* |
| *Median* | 16mn53s | 14h20mn | **12h36mn** |
| *Minimum* | 6mn21s | 10h04mn | 10h06mn |
| *Maximum* | 50mn55s | 7days 22h | 20h50mn |
|  |  |  |  |
| Table 2 Comparison of the performances of the three integration schemes for simulation 2. a) Mean absolute (and relative) error for estimates of forward and backward modulation. b) Computation time obtained during the fit. | | | |

Using DDE_RK, RelTol=1% saves 12% of computational time and the gain is less pronounced than in simulation 1 (38%). During the fit, the maximum number of iterations was not limited to guarantee the convergence of the variational optimization scheme (default criterion of convergence being 4 successive variations of free energy smaller to 1e-1) and this may sometimes cause a huge amount of time (in particular when fitting the *false* model B to data generated with a model FB).

## Application: Mismatch negativity EEG dataset

The seven models (see Fig. 3, main text) were fitted to the MMN dataset using DDE_RK, RelTol=1% and the model B was identified as the winning model with a posterior probability p=0.87 (see Fig. 4). This inference differed from the one made using either DDE_TA or DDE_RK, RelTol=0.1% which identified the same model FBI as the winning model. However, the use of DDE_RK, RelTol=1% resulted, for each model, in a higher relative free energy, when compared to the use of DDE_TA.

Despite the inconsistency of the Bayesian model comparison (which may ensue from the fact that here, one single dataset is used instead of many subjects as this is the case in DCM group studies), we compared the estimates of parameters for the model FBI done by the three integration schemes (see Fig. 4b). Similarities between DDE_RK, RelTol=0.1% and DDE_RK, RelTol=1% were especially more pronounced in the estimation of the modulation and conduction delay of the forward connections 1->3 and 2->4.

| 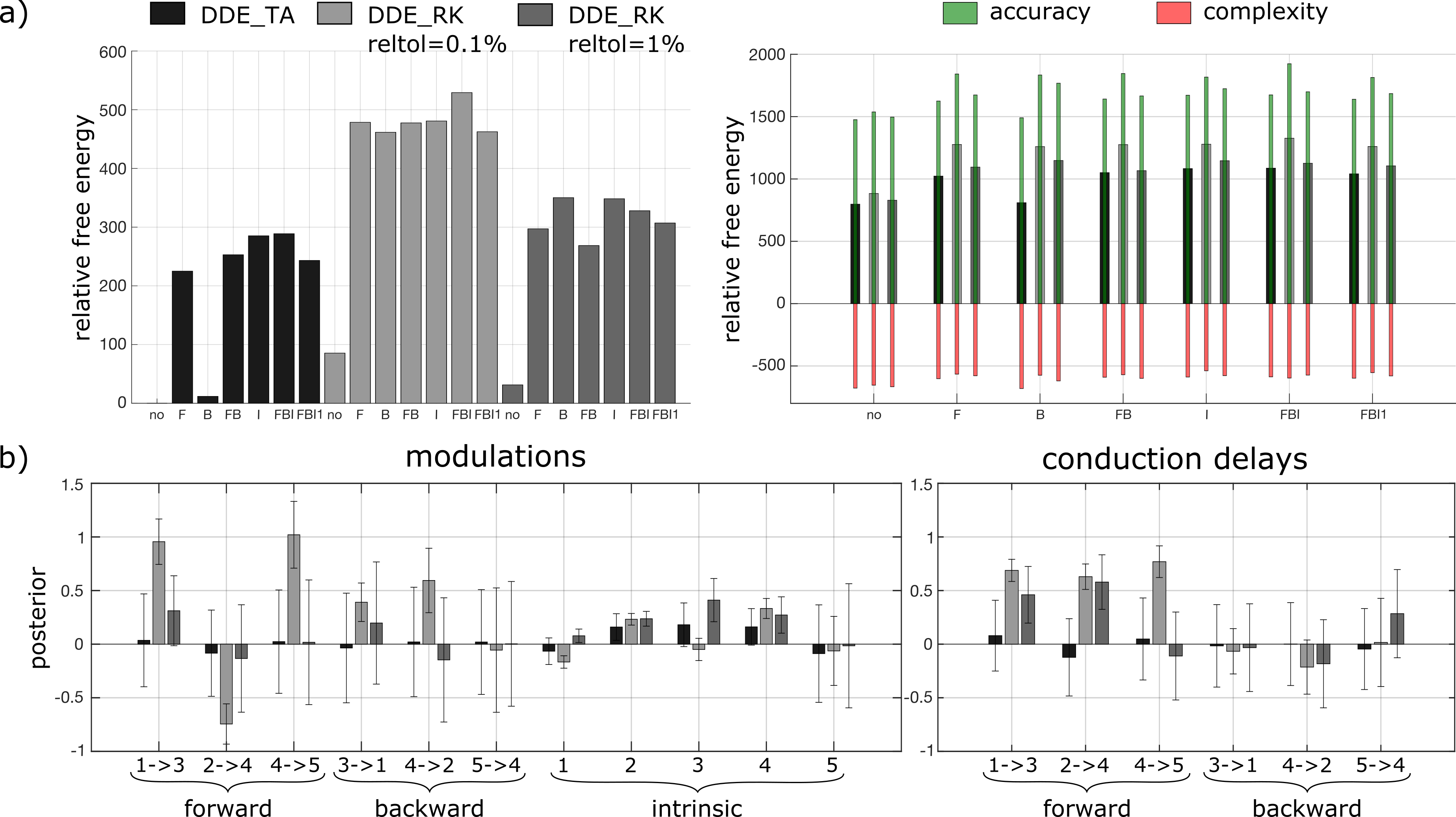 |
| --- |
| Fig. 4 DCM results on a MMN dataset. a) Left: Comparison of the relative free energy between the 7 tested models for DDE_TA (in black), DDE_RK, RelTol=0.1% (in light gray) and DDE_RK, RelTol=1% (in dark gray). Right: Decomposition of the relative free energy into accuracy (green) and complexity (red). b) Posterior estimation of forward, backward and intrinsic modulations (left plot) and conduction delays (right plot) with their 90% credibility interval for the model FBI. |

Fitting the MMN dataset with DDE_RK, RelTol=1% saved 52% of computational time (see Table 3).

| *Computation time* | | | |
| --- | --- | --- | --- |
|  | *DDE_TA* | *DDE_RK, RelTol=0.1%* | *DDE_RK, RelTol=1%* |
| *Median* | 4mn30s | 6h05mn | **2h56mn** |
| *Minimum* | 3mn14s | 2h13mn | 1h41mn |
| *Maximum* | 5mn34s | 21h25mn | 7h28mn |
| Table 3 Computational time for the fit of the MMN dataset presented for the three integration schemes. | | | |

# Conclusion

Using a smaller step size of 0.1ms for DDE_TA had no effect on the accuracy of the integration and was therefore not considered further. On the other hand, increasing the relative error tolerance of DDE_RK up to 1% appeared to be a good alternative to save computational time, at the cost of a limited inaccuracy.

Indeed, simulations 1 and 2 exhibited a very similar Bayesian model comparison and a very slight loss of precision for the estimation of parameters. Fitting the single MMN dataset using DDE_RK with a relative error tolerance of 1% also resulted in a systematic higher relative free energy for each of the seven models, compared to DDE_TA. The different ranking of the models is difficult to interpret here because a single dataset is used for the BMC (instead of 80 in the first simulation and 100 in the second simulation) and it is therefore more sensitive to the presence of local minima in the variational optimization procedure.

Finally, all the models used to fit the (simulated or real) data systematically obtained a higher evidence with DDE_RK (with either reltol =0.1% or 1%) compared to DDE_TA. And, for the MMN dataset, a higher relative error tolerance of 1% (instead of the default more stringent 0.1%) controlled by the DDE_RK integration scheme saved 52% of computational time.
